# The effect of COVID-19 pandemic and lockdown on consultation numbers, consultation reasons and performed services in primary care: results of a longitudinal observational study

**DOI:** 10.1186/s12875-021-01471-3

**Published:** 2021-06-23

**Authors:** Ingmar Schäfer, Heike Hansen, Agata Menzel, Marion Eisele, Daniel Tajdar, Dagmar Lühmann, Martin Scherer

**Affiliations:** Department of Primary Medical Care, University Medical Center Hamburg-Eppendorf, Martinistr. 52, 20246 Hamburg, Germany

**Keywords:** Covid-19, Primary care, Health care utilisation

## Abstract

**Objectives:**

The aims of our study were to describe the effect of the COVID-19 pandemic and lockdown on primary care in Germany regarding the number of consultations, the prevalence of specific reasons for consultation presented by the patients, and the frequency of specific services performed by the GP.

**Methods:**

We conducted a longitudinal observational study based on standardised GP interviews in a quota sampling design comparing the time before the COVID-19 pandemic (12 June 2015 to 27 April 2017) with the time during lockdown (21 April to 14 July 2020). The sample included GPs in urban and rural areas 120 km around Hamburg, Germany, and was stratified by region type and administrative districts. Differences in the consultation numbers were analysed by multivariate linear regressions in mixed models adjusted for random effects on the levels of the administrative districts and GP practices.

**Results:**

One hundred ten GPs participated in the follow-up, corresponding to 52.1% of the baseline. Primary care practices in 32 of the 37 selected administrative districts (86.5%) could be represented in both assessments. At baseline, GPs reported 199.6 ± 96.9 consultations per week, which was significantly reduced during COVID-19 lockdown by 49.0% to 101.8 ± 67.6 consultations per week (*p* < 0.001). During lockdown, the frequency of five reasons for consultation (-43.0% to -31.5%) and eleven services (-56.6% to -33.5%) had significantly decreased. The multilevel, multivariable analyses showed an average reduction of 94.6 consultations per week (*p *< 0.001).

**Conclusions:**

We observed a dramatic reduction of the number of consultations in primary care. This effect was independent of age, sex and specialty of the GP and independent of the practice location in urban or rural areas. Consultations for complaints like low back pain, gastrointestinal complaints, vertigo or fatigue and services like house calls/calls at nursing homes, wound treatments, pain therapy or screening examinations for the early detection of chronic diseases were particularly affected.

## Background

The COVID-19 pandemic originated from China and spread across the world during the year 2020. By mid-October 2020 more than 40,000,000 people worldwide had been tested positive for SARS-CoV-2 and more than 1,100,000 infected patients had died [1]. With more than 360,000 confirmed COVID-19 infections, corresponding to a cumulative incidence of 3.5%, and more than 9,700 COVID-19-associated deaths, the pandemic had had moderate impact on the population in Germany until this time [2].

As political reaction to the COVID-19 pandemic in Germany, a lockdown of many aspects of the social life was enforced from 13 March on. The lockdown included that – amongst other measures – all schools and facilities for childcare [3], education, sport, recreation and amusement had to close [4], contact restrictions for all people within Germany were decreed [4], planned hospital admissions and operations had to be postponed if medically justifiable [5] and visitations in hospitals and nursing homes were strictly regulated [6]. From early May to end-June, most of these measures were ended, albeit in small steps and inconsistently across the federal states of Germany. For example, in the federal state of Lower Saxony, on 6 May, hairdressers and shops with less than 800m^2^ sales floor were allowed to open under strict regulations, but it was not before 22 June until childcare facilities and schools were opened for all children again [7].

For most patients in Germany who had contact with infected people, returned from areas with a high COVID-19 prevalence, or suffered from mild to moderate symptoms related to COVID-19, the general practitioner (GP) was the first medical contact in the healthcare system. From the beginning, the COVID-19 pandemic was challenging for many GP practices in Germany. In order to protect the medical personnel and their patients, protective clothing, equipment, and disinfectants had to be obtained from a tight market and possibly infected patients had to be separated from patients without presumed COVID-19 infection [8]. Similar problems were also reported in primary care of other European countries [9, 10].

The contact restrictions and social isolation during lockdown may have led to adverse health outcomes in patients, eg, there were studies describing a reduction of physical activity [11], an increase in alcohol consumption [12] and a higher risk for psychiatric disorders [13]. The time during lockdown probably also saw a major change in the utilisation behaviour of the patients. On the one hand, inpatient and outpatient healthcare utilisation seemed to be reduced. For example, Mauro et al. described that many patients from Italy having already scheduled a consultation with outpatient physicians did not attend for fear of leaving their home. The authors found that emergency department admissions decreased by more than 50% during March and April 2020 as compared to the time between December 2019 and February 2020 [14]. On the other hand, there were reports of patients hoarding medications and other medical equipment. For example, Kostev and Lauterbach maintained that the COVID-19 lockdown in Germany was associated with largely increased pharmacy purchases including psychotropic, neurological, and cardiovascular drugs [15].

Until now, there have been few studies investigating how the patients’ utilisation of primary care for other reasons than COVID-19 changed during the lockdown in comparison to the time before the pandemic. The aims of our study therefore were to describe the effect of the COVID-19 lockdown on primary care in Germany regarding 1) the number of consultations 2) the prevalence of specific reasons for consultation presented by the patients and 3) the frequency of specific services performed by the GP.

## Methods

Our study was based on the project “Outpatient Healthcare Research North (*Ambulante Versorgungsforschung Nord – AVFN*)”, which aimed to explore regional variations in primary care of Northern Germany from the GPs’ and patients’ perspectives [16]. In order to monitor the impact of the COVID-19 pandemic and lockdown on primary care, a follow-up survey was conducted using the same study participants as in the original interviews. The baseline assessment was described in the study register ClinicalTrials.gov (NCT02558322), in the published study protocol [16] and a paper presenting results from the GP interviews [17]. In the following study, only GPs were included that completed both, the baseline and the follow-up assessment.

### Study design

For our survey, a quota sampling design was chosen to represent as many individual administrative districts and regionally different healthcare situations in the study area as possible. The purpose of this design was to raise the probability of also including underserved regions into the study where usually many GPs were unwilling to participate in a study due to their heavy workload. Details of the sampling procedure, the GP recruitment and the data collection at baseline can be found elsewhere [16, 17].

In short, all administrative districts were included in the study where at least 20% of the land area was located within a radius of 120 km (ca. 75 miles) around the study centre. We stratified the sample a) by regional category and b) within the regional categories by counties and independent cities proportionally to the respective population size in each district. The thus chosen administration districts for the study were derived from the German Federal States of Bremen, Hamburg, Mecklenburg-Western Pomerania, Lower Saxony, Saxony-Anhalt and Schleswig–Holstein [16].

### Recruitment of participants

GPs’ recruitment was based on a database of the Department and Policlinic of Primary Care at the University Medical Center Hamburg-Eppendorf as well as on the databases of the respective regional associations of statutory health insurance physicians in the selected areas. GPs were eligible for the study if they had an accreditation as statutory health insurance physician in the respective administrative district and used an EDP system facilitating to draw up a list of all patients treated over the preceding quarter (3-month accounting period) [16].

Eligible GPs were contacted in writing by mail and asked to participate in the study. In the letter, we provided general information about aims of the project and content of the questionnaire (eg, that we wanted to assess the frequency of reasons for consultations and services), but the GPs did not receive a detailed list of items in advance. Participating GPs gave informed consent and were subsequently visited by a staff member of the project and personally interviewed at baseline.

During COVID-19 lockdown, all GPs with a completed baseline assessment were contacted again by mail and asked for participation in the follow-up. In case of non-response, a) a reminder was sent by mail 14 days after the first letter; b) we tried up to five times to contact non-participating GPs by telephone 14 days after the postal reminder; and c) an email was sent 14 days after the telephone calls were finished. Due to contact restrictions during lockdown, the follow-up assessment was conducted as a postal survey. In both waves of data collection, GPs were allowed to check their medical records if they considered it necessary.

### Data set

The interviews were based on standardised questionnaires and contained information regarding the GPs’ age, sex, workload and postgraduate medical training as well as data on the practice. In Germany, GPs work mostly self-employed in individual private practices. If physicians choose to work together with other physicians in Germany, they can do it self-employed in private practices where each physician submits his own claims (group practice) or in private practices where the claims of all physicians are combined and submitted as one bill (joint practice). It is also possible that GPs work as employees or self-employed in legally independent care facilities with several physicians called medical care centres (“*Medizinische Versorgungszentren*”).

Additionally, we documented consultation reasons (“How often (per day/week/month) do you see patients with the following reasons for consultations?”) and healthcare services (“How often (per day/week/month) do you provide the following services?”). This assessment was based on a standardised instrument developed on the basis of the International Classification of Primary Care (ICPC-2) [16, 18, 19]. Additionally, the baseline interview contained questions about the number of weekly contacts with 27 patient types, which have been reported elsewhere [20]. The follow-up assessment of consultation reasons and services included all categories with a frequency ≥ 1.0% at baseline and questions about the effect of COVID-19 on primary care in the selected practices. The resulting data were described as frequency per week.

Initially, regional comparisons had been based on the three categories “urban areas”, “environs” and “rural areas”. The analysis of the baseline data did show, however, that the middle category “environs” was of little explanatory value [17]. We therefore decided to redefine the regional variable for the study presented here and to use only two categories for all included administrative districts. The regional categories were defined according to the so-called “structural settlement of district types” of the German Federal Institute for Research on Building, Urban Affairs and Spatial Development [21]. The category “urban areas” included urban municipalities (with more than 100,000 inhabitants in total) and urbanised districts (with more than 300 inhabitants/km^2^), and the category “rural areas” included rural districts with signs of agglomeration (with more than 150 inhabitants/km^2^) and sparsely populated rural districts (with less than 150 inhabitants/km^2^).

Thus, the study included under the category “urban areas” the administration districts Bremen, Hamburg, Harburg, Kiel, Lübeck, Osterholz, Pinneberg and Stormarn. The category “rural areas” comprised the administration disctricts Altmarkkreis Salzwedel, Celle, Cuxhaven, Delmenhorst, Diepholz, Dithmarschen, Gifhorn, Heidekreis, Herzogtum-Lauenburg, Ludwigslust-Parchim, Lüchow-Dannenberg, Lüneburg, Neumünster, Nienburg (Weser), North Frisia, Northwestern Mecklenburg, Oldenburg, Ostholstein, Plön, Rendsburg-Eckernförde, Rotenburg (Wümme), Schleswig-Flensburg, Schwerin, Segeberg, Stade, Steinburg, Uelzen, Verden and Wesermarsch.

### Statistical analyses

Descriptive data were presented as percentages and as means and standard deviations, respectively. Differences between the time before the COVID-19 pandemic and the time during lockdown were described using the t-test. The association between the COVID-19 pandemic and lockdown and the number of consultations was subsequently analysed by multivariate linear regressions in mixed models adjusted for random effects on the levels of administrative districts and GP practices within the districts. In these analyses, the control variables were stepwise included in three statistical models: 1) a naïve comparison adjusted for differences between administrative districts, the time since lockdown on 13 March 2020 and the time between the baseline and the follow-up assessment; 2) model 1 additionally adjusted for age, sex, and the postgraduate medical specialist training of the GP; and 3) model 2 additionally adjusted for the type of practice. A potential improvement of the model fit was determined by the likelihood ratio test.

An alpha level of 5% (*p *≤ 0.05) was defined as statistically significant for all analyses of the inferential statistics. In order to address multiple testing, analyses were adjusted for the familywise error rate using the Benjamini–Hochberg procedure [22]. Analyses of consultation reasons were adjusted for 29 statistical tests and analyses of the performed services were adjusted for 22 statistical tests. As the number of consultations was analysed by three statistical tests in nested models, no adjustment had to be performed for these analyses. Stata 15.1 was used for data preparation and data analysis.

## Results

In the baseline assessment, 211 GPs had participated from 12 June 2015 to 27 April 2017. Detailed results of the initial GP recruitment are described elsewhere [17]. Between baseline and follow-up, 19 GPs (9.0%) had retired, 38 GPs (18.0%) refused to participate in the follow-up and 44 GPs (20.9%) did not respond to our invitation to the second assessment. Finally, 110 GPs (52.1%) participated in the follow-up between 21 April 2020 and 14 July 2020. Of those, 104 questionnaires (94.6%) were returned during the first month and only 6 questionnaires were sent back after 20 May 2020. Primary care practices in 32 of the 37 selected administrative districts (86.5%) could be represented in both assessments. No follow-up data are available for four rural districts (Delmenhorst, Diepholz, Herzogtum Lauenburg and Schleswig-Flensburg) and one urban district (Osterholz).

The participating GPs had a mean age of 53.7 ± 7.9 years and 63.6% were male. 84.6% of the GPs had postgraduate training as specialist in general practice and 21.8% had postgraduate training as specialist in internal medicine. 1.8% of GPs reported that they had not completed a postgraduate specialist training. In our study, 54.6% of the GPs worked in individual practices, 8.2% in group practices (“*Praxisgemeinschaften*”) and 35.5% in joint practices (“*Gemeinschaftspraxen*”). Additionally, 1.8% of the GPs worked in medical care centres (“*Medizinische Versorgungszentren, MVZ*”). The GPs reported to have a working time of 45.6 ± 12.1 h per week at baseline, which was reduced by 21.6% to 35.9 ± 13.8 h per week during lockdown. The difference in working time was statistically significant (*p *< 0.001).

During lockdown, 45.9% of the GPs reported that employees of their practice had to stop working temporarily because of reasons related to COVID-19, eg, because they were in quarantine or infected with SARS-Cov-2. In 34.9% of the practices, 3.1 ± 7.9 medical assistants were absent for 14.4 ± 15.1 working days. In 19.3% of the practices, 1.7 ± 1.2 physicians were absent for 9.1 ± 6.0 working days. Additionally, 6.4% of the practices had to close for 5.3 ± 4.7 working days due to reasons related to COVID-19.

At baseline, GPs reported 199.6 ± 96.9 consultations per week, which was significantly reduced during COVID-19 lockdown by 49.0% to 101.8 ± 67.6 consultations per week (*p *< 0.001). The multilevel, multivariable analyses are shown in Table 1. They confirm that there was a significant effect of COVID-19 pandemic and lockdown. In all three statistical models, pandemic and lockdown were associated with an average reduction of 94.1 to 94.6 consultations per week. This effect was independent of the time since lockdown and without significant difference between urban and rural areas. The inclusion of age, sex and the postgraduate specialist training of the GP did neither improve the model fit (*p *= 0.291) nor change the effect estimated in the nested model described above. The model fit improved significantly (*p *= 0.016) after inclusion of the type of practice the GP worked in, but there was practically no change in coefficient and *p-*value of the difference between baseline and follow-up. However, in comparison to the consultation numbers reported by GPs working in individual practices, the number of consultations reported by GP working in group practices (-45.3 consultations per week) and joint practices (-35.2 consultations per week) was even further reduced.

**Table 1:** Association between covid-19 lockdown and the number of treated patients: results of a multivariate linear regression adjusted for random effects on the levels of German federal states, administrative districts within the federal states and GP practices

	**Model 1**		**Model 2**		**Model 3**	
	**ß (95% CI)**	***p***	**ß (95% CI)**	***p***	**ß (95% CI)**	***p***
***Assessment during covid-19 lockdown compared to assessment before covid-19***	***-94.6 (-116.7 to -72.5)***	** < *0.001***	***-94.6 (-116.2 to -73.0)***	** < *0.001***	***-94.1 (-115.3 to -73.0)***	** < *0.001***
Days since lockdown on 13 March 2020	-0.34 (-1.3 to 0.57)	0.466	-0.30 (-1.2 to 0.61)	0.517	-0.29 (-1.2 to 0.60)	0.522
Days between baseline and follow-up	0.043 (-0.017 to 0.10)	0.160	0.041 (-0.021 to 0.10)	0.193	0.061 (-0.0018 to 0.12)	0.057
Region: urban areas vs. rural areas	13.5 (-8.9 to 35.8)	0.238	12.1 (-11.4 to 35.6)	0.312	6.1 (-18.2 to 30.3)	0.622
Age of the physician (in years)			0.99 (-0.47 to 2.4)	0.184	0.58 (-0.87 to 2.0)	0.434
Sex of the physician: male vs. female			10.4 (-13.9 to 34.7)	0.400	14.0 (-9.8 to 37.8)	0.249
Postgraduate medical specialist training						
- None (general practitioner)			-39.0 (-131.9 to 53.9)	0.410	-53.8 (-146.2 to 38.7)	0.254
- General medicine			23.3 (-21.3 to 68.0)	0.305	20.6 (-23.2 to 64.3)	0.357
- Internal medicine			10.3 (-28.2 to 48.7)	0.601	9.5 (-27.9 to 46.9)	0.617
***Type of practice***						
***- Group practice vs. individual practice***					***-45.3 (-87.0 to -3.6)***	***0.033***
***- Joint practice vs. individual practice***					***-35.2 (-59.3 to -11.1)***	***0.004***
- Medical care centre vs. individual practice					8.2 (-73.3 to 89.8)	0.843

Model 1: naïve comparison adjusted for differences between administrative districts, the time since lockdown on 13 March 2020 and the time between baseline and follow-up; model 2: model 1 additionally adjusted for age, sex, and postgraduate medical specialist training of the GP; model 3: model 2 additionally adjusted for the type of practice; ß: ß correlation coefficient; 95% CI: 95% confidence interval; *p*: *p-*value

During lockdown, the frequency of 18 reasons for consultation was reduced and the frequency of 11 reasons for consultation had increased (cf. Table 2). The highest reduction was found in the category “adverse drug reaction” (-50.5%) and the highest increase was found in the category “workplace issues, unemployment” (+ 90.2%). However, due to the large variance between GP practices these differences were not statistically significant after the Benjamini-Hochberg-adjustment. A statistically significant reduction was found in the categories “total gastrointestinal tract” (-43.0%), “upper gastrointestinal tract” (-36.6%), “vertigo” (-35.7%), “spinal disorders” (-34.6%) and “general fatigue and weakness” (-31.5%).

**Table 2:** Change between baseline and follow-up in the frequency per week of reasons for consultation^a^

	**Baseline**	**Follow-Up**	**Change**	***p***	**p**_**adj**_^b^
Adverse drug reaction	8.5 ± 22.0	4.2 ± 4.7	-50.5%	0.054	0.157
***Total gastrointestinal tract***	***17.6* ± *19.7***	***10.0* ± *13.4***	***-43.0%***	***0.001***	***0.041***
Lipid metabolism disorder	22.0 ± 37.3	13.7 ± 24.1	-37.6%	0.059	0.156
Lower gastrointestinal tract	11.7 ± 15.2	7.4 ± 14.6	-36.9%	0.038	0.157
Weight/dietary problems	19.5 ± 45.6	12.3 ± 35.3	-36.8%	0.205	0.397
***Upper gastrointestinal tract***	***16.1* ± *16.1***	***10.2* ± *11.5***	***-36.5%***	***0.003***	***0.041***
***Vertigo***	***11.5* ± *12.2***	***7.4* ± *7.3***	***-35.7%***	***0.004***	***0.027***
***Spinal disorders***	***34.8* ± *29.1***	***22.8* ± *28.4***	***-34.6%***	***0.003***	***0.028***
High blood pressure	40.0 ± 45.8	26.9 ± 44.1	-32.8%	0.037	0.176
Somatoform disorders	22.3 ± 26.2	15.3 ± 23.7	-31.6%	0.043	0.156
***General fatigue and weakness***	***20.0* ± *19.4***	***13.7* ± *14.1***	***-31.5%***	***0.008***	***0.047***
Lymph/leg oedema, chronic wounds	6.9 ± 9.2	5.0 ± 7.2	-28.1%	0.090	0.218
Coronary heart disease	16.4 ± 21.5	12.0 ± 35.2	-26.7%	0.279	0.477
Congestive heart failure	12.1 ± 16.9	8.9 ± 16.7	-26.3%	0.175	0.390
Cardiac arrhythmia	12.0 ± 18.5	9.3 ± 10.0	-22.3%	0.194	0.402
Dementia	8.4 ± 15.3	6.8 ± 11.8	-19.2%	0.393	0.569
Diabetes mellitus (all types)	21.9 ± 26.3	17.7 ± 27.6	-19.2%	0.263	0.477
Thyroid disorder	10.2 ± 17.3	9.5 ± 23.4	-6.0%	0.832	0.862
Arthrosis of the large joints	9.6 ± 9.2	9.7 ± 14.1	1.9%	0.914	0.914
Shoulder pain	6.6 ± 6.7	6.9 ± 9.0	5.2%	0.756	0.812
Acute infections of the respiratory tract	40.2 ± 39.1	42.6 ± 45.9	5.9%	0.686	0.795
Mild mental disorders	15.3 ± 18.5	16.5 ± 28.2	7.5%	0.730	0.814
Headache	9.2 ± 9.3	10.3 ± 13.6	11.9%	0.497	0.626
Urinary tract infection	5.8 ± 4.4	6.7 ± 7.6	15.4%	0.296	0.476
Chronic obstructive pulmonary disease	7.3 ± 9.1	8.7 ± 15.3	19.5%	0.420	0.580
Family, partnership problems	9.1 ± 16.2	11.2 ± 22.2	24.0%	0.421	0.555
Chronic kidney disease	5.7 ± 15.4	7.1 ± 21.2	24.5%	0.588	0.711
Asthma	6.3 ± 9.9	8.1 ± 15.0	27.4%	0.326	0.497
Workplace issues, unemployment	7.0 ± 9.3	13.3 ± 30.3	90.2%	0.045	0.144

^a^ sorted in ascending order by change between baseline and follow-up; ^b^ α-level Benjamini-Hochberg-adjusted for 29 statistical tests; statistically significant results in bold and italic

The GPs reported that 53.6% of the reason “acute infections of the respiratory tract” were related to COVID-19. Every week, the GPs treated 17.6 ± 21.6 patients who consulted the GP because of fear of a COVID-19 infection, 5.1 ± 8.3 patients who had returned from a COVID-19 risk area or had been in close contact with a confirmed COVID-19 patient and 0.64 ± 0.78 patients who received a confirmed COVID-19 diagnosis. Additionally, 33.3% of the GP reported that they had to care for 6.4 ± 14.5 patients being in domestic quarantine related to COVID-19.

During lockdown, the frequency of 18 services had decreased and four services had been performed more frequently than before the COVID-19 pandemic (cf. Table 3). The highest reduction was found in the category “house calls and calls at nursing homes” (-56.6%; *p *= 0.001). Other statistically significant differences were found in the categories “stool examination” (-49.4%), “referral to specialist” (-47.6%), “check-up 35” (-46.3%) and eight other categories. The highest increase in frequency was found in the service category “organisation of patient care/support” (+ 28.5%), but this difference was not statistically significant.

**Table 3:** Change between baseline and follow-up in the frequency per week of performed services^a^

	**Baseline**	**Follow-Up**	**Change**	**p**	**p**_**adj**_^b^
***House calls (also: calls at nursing homes)***	***31.6* ± *54.1***	***13.7* ± *17.3***	***-56.6%***	***0.001***	***0.002***
Local injection/infiltration	16.6 ± 43.2	7.7 ± 11.1	-53.5%	0.039	0.061
***Stool examination***	***10.4* ± *13.5***	***5.2* ± *6.6***	***-49.4%***	***0.001***	***0.004***
***Referral to specialist***	***93.4* ± *89.0***	***49.0* ± *52.9***	***-47.6%***	** < *0.001***	** < *0.001***
***Check-up 35***^c^	***12.8* ± *12.6***	***6.9* ± *8.3***	***-46.3%***	** < *0.001***	***0.001***
Physical therapy	9.2 ± 16.9	5.1 ± 11.5	-45.1%	0.037	0.063
***Urine analysis***	***39.5* ± *26.4***	***22.0* ± *17.2***	***-44.4%***	** < *0.001***	** < *0.001***
***Pain therapy***	***20.2* ± *30.9***	***11.5* ± *24.2***	***-43.2%***	***0.022***	***0.041***
***Skin cancer screening***	***8.4* ± *11.6***	***4.9* ± *7.4***	***-41.8%***	***0.010***	***0.025***
***Electrocardiogram (ECG)***	***20.7* ± *17.4***	***12.5* ± *11.1***	***-39.7%***	** < *0.001***	** < *0.001***
***Blood tests***	***87.5* ± *61.9***	***52.8* ± *32.8***	***-39.7%***	** < *0.001***	** < *0.001***
Nutrition counselling	16.7 ± 27.0	10.3 ± 24.1	-38.0%	0.070	0.103
***Pulmonary function test***	***9.5* ± *10.9***	***5.9* ± *9.4***	***-37.5%***	***0.011***	***0.024***
***Sonography***	***14.5* ± *17.3***	***9.1* ± *12.2***	***-37.4%***	***0.008***	***0.023***
***Wound dressing/compression/tamponade***	***16.9* ± *19.8***	***11.3* ± *11.3***	***-33.5%***	***0.011***	***0.022***
Disease management programme (DMP)	21.0 ± 20.1	17.3 ± 19.5	-17.7%	0.181	0.249
Psychosomatic basic care	24.2 ± 26.7	22.3 ± 27.1	-7.8%	0.622	0.652
Vaccination	16.1 ± 14.2	15.0 ± 13.8	-7.1%	0.563	0.652
Social medical assessments	4.8 ± 4.7	5.1 ± 6.3	6.3%	0.689	0.689
Lifestyle counselling, social counselling	36.8 ± 53.1	39.9 ± 46.5	8.4%	0.616	0.677
Continuous care for the chronically ill	82.8 ± 78.8	99.4 ± 239.4	20.0%	0.479	0.585
Organisation of patient care/support	8.2 ± 10.9	10.5 ± 16.4	28.5%	0.200	0.259

^a^ sorted in ascending order by change between baseline and follow-up; ^b^ α-level Benjamini-Hochberg-adjusted for 22 statistical tests; statistically significant results in bold and italic; ^c^ From the age of 35, the health insurance pays a regular health check-up every three years. The check-up 35 comprises anamnesis (particularly regarding the risk profile), physical examination (including measurement of blood pressure), blood examination (including lipid profile and glucose), urine examination (including protein, glucose, red and white blood cells, nitrite), medical advice concerning the test results and – if medically indicated – prevention recommendations [23]

## Discussion

### Statement of principal findings

During COVID-19 pandemic and lockdown, we observed a dramatic reduction of the number of consultations of each attending GP. This effect was independent of age, sex and specialty of the GP and independent of the practice location in urban or rural areas. The decreased number of consultations was accompanied by fewer consultation for complaints like low back pain, vertigo or fatigue which – from a professional perspective – usually do not require urgent treatment and for which objective medical explanations are often difficult to find. The number of acute infections of the respiratory tract was comparable to the number assessed before lockdown. The time of mid-April in which the data collection was performed, however, is no usual season for respiratory infections, so there might be a hidden increase in these consultation reasons.

The analysis of the provided services indicated that the number of wound treatments and pain therapy had been significantly decreased by more than 30% and 40%, respectively. House calls and calls at nursing homes as well as referrals to specialists had been (almost) cut by half. Screening examinations for the early detection of chronic diseases like skin cancer, colon cancer (by stool examination) and diabetes or kidney diseases (“check-up 35”) had been largely reduced. And diagnostic procedures like blood or urine tests, electrocardiograms, sonography or lung function tests were also decreased by large numbers.

### Strengths and limitations

As our study was based on survey data, we were able to conduct analyses which would not have been possible if we used routine data. For example, in Germany, most services provided by GPs are paid by flat rate and therefore the quantity in which specific services are provided in primary care cannot be analysed using routine data. In contrast, services could be depicted individually in our study, and consultation reasons, such as “social problems”, for which no specific ICD-10 exist, could be assessed. Additional strengths were provided by our statistical methods which take possible confounders and the clustering of the dataset into consideration. In the analyses of reasons for consultations and provided services we adjusted the α-level for the familywise error rate in order to correct our analyses for multiple testing and to present a conservative view on the effects of pandemic and lockdown on primary care in Northern Germany.

GPs’ recruitment was based on a quota sampling and not on random participant selection. At follow-up, 86.5% of the selected administrative districts were still represented in our study. By stratifying our sample by administrative districts we could represent a) medically undersupplied and overserved districts and b) socioeconomically deprived and undeprived regions in our data set. Although random selection is a more frequently used and better known method, studies have shown that quota sampling does not necessarily result in a larger selection bias than random sampling [24]. A non-responder analysis revealed only minor deviations in the mean age between participating GPs and the total population in the selected regions (54.8 years vs. 53.7 years). The proportion of male GPs, however, was noticeably higher in our study (63.6% vs. 56.7%) [25]. But as there was no significant effect of the GPs’ sex on the number of consultations in our analyses, we are confident that these sex differences do not bias our analyses.

As in all studies based on self-reported data, the physicians’ answers might have been influenced by memory gaps, errors or social desirability. The assessment of the consultation numbers, the consultation reasons and the service spectrum was mainly retrieved from memory and they could not be verified through a clinical audit of records. Due to contact restrictions during the lockdown, the follow-up assessments could not be performed by personal interviews as at baseline. Instead, we decided to conduct a postal survey. The change in the methods for data collection might have affected the comparability between baseline and follow-up, eg, if GPs took more time at follow-up to think about their answers than they took at baseline. However, the GPs received the same questionnaire at baseline and follow-up (cf. additional file 1). We are therefore confident that – although the absolute numbers may be impacted by the same detection bias – the estimate of change is still valid.

It also needs to be mentioned that the mean time between both waves was 4.1 ± 0.51 years. This long time span might account for some of the differences between baseline and follow-up, eg, if GPs decided to reduce their workload due to increasing age. Some differences between baseline and follow-up might also by explained by changes in health policy. For example, the screening interval of the service “check-up 35” was changed in October 2019 from two years to three years [26], which probably caused a reduction in contacts due to this service in 2020.

Another limitation is the fact that the recruitment area has been restricted to Northern Germany and the results therefore do not necessarily represent the remainder of Germany. As this was an observational study with multiple outcomes, we were unable to carry out a sample size calculation and therefore we will probably have missed some effects of the lockdown. Loss to follow-up, which lowered the number of participating practices by 47.9%, further reduced the statistical power of our analyses and also might have affected the representativeness of our study.

### Comparison with the literature

Few other studies have analysed the impact of the COVID-19 pandemic during lockdown on primary care. In a qualitative study from Flanders with 132 participating GPs, the study participants reported that patients consulted their GP less frequently for reasons other than COVID-19 as compared to the time before the pandemic. The GPs expressed the feeling that acute care was compromised, chronic disease care was mostly postponed and that consequences will be visible after the pandemic [27].

A retrospective cohort study based on routinely collected primary care data in the UK found a 50.0% reduction in the first diagnoses of common mental health problems, a reduction of 49.0% in type 2 diabetes diagnoses and a reduction of 43.3% in diagnoses of circulatory system diseases between March and May 2020. In the same time period, first prescriptions of associated medications were also significantly lower than expected [28].

A study from Spain based on quality indicators in primary care found a negative effect of COVID-19 pandemic and lockdown on the follow-up in patients treated with anticoagulants and patients with diabetes, hypertension, chronic ischemic heart disease and cerebrovascular accidents. They also reported a largely reduced number of screenings, particularly diabetic foot screening and diabetic retinopathy screening, and some vaccination indicators like Measles-Mumps-Rubella were also affected [29].

There are also many data from the hospitals’ emergency departments which indicate similar changes in consultation numbers as in our study. For example, a study analysing more than 1,000,000 emergency department visits in Germany stated that the number of cases during the COVID-19 pandemic decreased by up to 38% compared to the previous year [30]. Other studies came with similar results [31] or described a largely reduced number of cases in inpatient urological [32], rheumatological [33] and paediatric care [34], in hospital admissions due to ischemic cerebrovascular events [35], heart failure and cardiac arrhythmias [36], and in ophthalmological emergency department visits [37]. However, it should be noted that German hospitals were instructed by policy to postpone non-urgent medical interventions to keep beds for severely ill COVID-19 patients [5]. There was no such political instruction for primary care.

### Implications for research and clinical practice

Our study documented the GPs’ perception that many patients did not utilise their practice during lockdown and that many important services have been provided in a largely reduced numbers. Other studies should examine if these results can be confirmed in other countries and with different designs. In particular, we need more information on the underlying reasons for the lower number of cases, ie, if patients refrained from utilising primary care during pandemic and lockdown or if the GPs asked the patients to postpone not urgently necessary consultations, eg, because of being afraid that patients without symptoms might carry COVID-19 into their practices. We also need to investigate which health problems were most affected from the patients’ perspective and what were their subjective reasons for not utilising primary care.

In our study, we also see large differences in absolute numbers between the time before COVID-19 and the time during lockdown, which are not confirmed by statistical means, but are in our view still worthwhile to reassess in future studies. Particularly, the following questions should be examined: Have patients with chronic respiratory diseases like asthma and COPD utilised their GP more often than before COVID-19, eg, in order to clarify if symptoms of dyspnoea are related to COVID-19 [38]? Have other high risk patients, eg, patients with coronary heart disease, congestive heart failure [39] or diabetes mellitus [40], missed consultations with their GP, eg, in fear of getting infected with COVID-19? And have patients with social problems related to the lockdown, eg, family or partnership problems [41] and unemployment or workplace problems [42, 43], consulted their GP more often than before the lockdown?

The largely reduced number of cases in primary care, the reduced prevalence of consultation reasons and the reduced number of provided services might point to unmet medical needs in general practice during pandemic and lockdown [27–29]. There are some other reasons that might affect these numbers and also need to be mentioned. It is possible, that changes in health behaviour, such as reducing social contacts or wearing a face mask, reduced the spread of other communicable diseases [44], such as gastrointestinal infections. It is also possible, that patient utilised medical services by telephone. For example, during lockdown, patients were allowed to contact their family doctor by telephone and, in the case of mild symptoms, to receive a notification of illness by mail [45]. Patients with COVID-19-related symptoms who did not have a family doctor or whose family doctor refused to treat infectious patients could contact a telephone hotline and receive an appointment in newly set-up "Infectious Disease Practices" or could be treated by a doctor at home [46].

At the same time, many GPs seem to have reduced home calls and calls at nursing homes. One reason for this finding might be a lack of protective equipment [8]. If the GPs could not protect themselves during patient consultations, they could not exclude to carry SARS-CoV-2 and might have refrained from entering a nursing home. The reduced number of calls at nursing homes could account for the reduction in specific services, especially the decrease in wound treatments. Patients in nursing homes are one of the most vulnerable patient groups with regard to COVID-19 and were at the same time at risk for adverse health outcome due to isolation during the lockdown [47]. The medical care of frail elderly living alone and patients in nursing homes should be ensured while the number of COVID-19 infections is increasing again.

Another finding that needs to be discussed is the reduced number of screenings for early detection of cancer and other chronic diseases. A reduced number of cancer diagnoses is also reported from the Netherlands [48] and the UK [49]. While referral of patients diagnosed with high risk cancer should never be delayed, a small delay in the diagnosis of low risk cancer probably only marginally affects the prognosis and quality of life of the patient [48]. However, GPs should be alert for symptoms of cancer and other chronic conditions whose diagnosis might be delayed due to the lockdown.

## Conclusions

We observed a dramatic reduction of the number of consultations in primary care. This effect was independent of age, sex and specialty of the GP and independent of the practice location in urban or rural areas. Consultations for complaints like low back pain, gastrointestinal complaints, vertigo or fatigue and services like house calls/calls at nursing homes, wound treatments, pain therapy or screening examinations for the early detection of chronic diseases were particularly affected. After lockdown, GPs should be alert for unmet medical needs, particularly in frail elderly living alone and patients in nursing homes. Additionally, GPs should encourage their patients to discuss symptoms of cancer and other chronic conditions, which might have been neglected during lockdown.

## Data Availability

The questionnaires are available in additional file 1. Study data cannot be shared, because it is not permitted by participant consent and ethics approval. However, data are available from the corresponding author on reasonable request.
